# Operational research in infectious disease outbreak response: An analysis of the World Health Organization’s Disease Outbreak News

**DOI:** 10.1371/journal.pgph.0005802

**Published:** 2026-07-02

**Authors:** Melissa Rosenthal, Jeni Stolow, Florian Vogt, Hannah Barnsley, Eleanor Caine, Daniela Garone, Gail Carson, Lauren Sauer, Victor Del Rio Vilas, Lucia Mullen

**Affiliations:** 1 Celia Scott Weatherhead School of Public Health and Tropical Medicine, Tulane University, Department of International Health and Sustainable Development, New Orleans, Louisiana, United States of America; 2 Celia Scott Weatherhead School of Public Health and Tropical Medicine, Tulane University, Department of Social Behavioral and Population Sciences, New Orleans, Louisiana, United States of America; 3 The Kirby Institute, University of New South Wales, Sydney, Australia; 4 National Centre for Epidemiology and Population Health, Australian National University, Canberra, Australia; 5 UK Public Health Rapid Support Team, UK Health Security Agency, London, United Kingdom; 6 UK Health Security Agency, London, United Kingdom; 7 Médecins Sans Frontières International, Brussels, Belgium; 8 University of Oxford, Oxford, United Kingdom; 9 Department of Environmental, University of Nebraska Medical Center, College of Public Health, Agricultural, and Occupational Health, Omaha, Nebraska, United States of America; 10 Johns Hopkins Center for Health Security, Baltimore, Maryland, United States of America; 11 Department of Environmental Health and Engineering, Johns Hopkins University Bloomberg School of Public Health, Baltimore, Maryland, United States of America; Mahidol University, THAILAND

## Abstract

Timely and effective response to infectious disease outbreaks depends on the rapid application of evidence-based policies. Operational research (OR) is conducted during emergency response to inform decision-making, and guide response strategies; however, there is no standardized mechanism for documenting how OR is conducted or communicated during outbreaks. The World Health Organization Disease Outbreak News (DONs) platform is a global reporting mechanism that provides publicly-accessible updates on acute public health emergency response activities. Although DONs reports describe outbreak response actions, the extent to which they capture OR has not been examined. This study reviewed DONs reports to characterize how outbreak response efforts are documented and to assess the visibility of OR reporting. A structured content analysis was conducted of DONs reports published between January 2012 and October 2024. Reports describing acute infectious disease outbreaks were included. Quantitative data were extracted on outbreak response activities, deployment, technical support, and OR. Reports were deductively coded using a coding framework to identify thematic patterns. 412 outbreak events were included. Most DONs reports described deployment activities (100%) and technical support (95.1%). OR was explicitly mentioned in only 9 reports (2.2%).. Thematic analysis identified four key patterns: increasing report completeness over time; frequent association of deployment with laboratory and diagnostic activities; frequent association of technical support with infection prevention and control; and inconsistent OR reporting. Terminology used to describe response varied across reports, making systematic identification of OR difficult. While DONs reports provide valuable and accessible information on outbreak response activities, they capture limited information on OR. Enhancing reporting guidance, including clearer terminology and OR documentation, could improve the ability to share lessons learned. While DONs are not intended as the sole forum for reporting OR, strengthening their role as a communication platform may support knowledge sharing and contribute to effective, evidence-informed outbreak response.

## Introduction

Rapid and effective response to infectious disease outbreaks relies on the timely application of evidence-based policies and practices. Operational research (OR) is often essential for generating the data needed to guide these actions and to facilitate coordination, communication, and decision-making across emergency response efforts, including outbreaks, natural disasters, and humanitarian crises. This work may involve piloting communication materials, testing implementation approaches in the field, or evaluating new vaccine delivery strategies [1,2].

Although there is broad agreement on the importance of operational research for strengthening response efforts, there is no universally accepted definition within the infectious disease outbreak response literature or the broader field of public health. A clear and consistent definition is needed to ensure that operational research can be systematically prioritized, funded, taught, supported, and communicated across emergency contexts, thereby improving the speed, quality, and effectiveness of outbreak, emergency, and humanitarian responses. However, developing such a definition is challenging because operational research is often conducted in fast-moving settings, where activities may be difficult to track, document, and formally classify. For the purposes of this manuscript, operational research is defined as the analysis of procedures and practices to inform decision-making and policy [2].

At the time of publication, no single repository exists to systematically capture and track the implementation of operational research during infectious disease outbreaks. To date, the closest available resource is the World Health Organization’s (WHO) Disease Outbreak News (DONs) platform, which routinely reports on outbreak events and response activities. Although the DONs were not designed to report operational research, they have historically discussed ongoing investigation and research activities [3].

The Disease Outbreak News (DONs) platform serves as the primary publicly available mechanism through which the World Health Organization (WHO) communicates information on acute public health emergencies. Established in 1996, DONs support WHO’s compliance with Article 11.3 of the International Health Regulations (IHR 2005), which requires Member States to share information with WHO regarding urgent outbreaks and implemented control measures [4,5]. DONs reports typically include information on incidence and prevalence, geographic distribution, recommended response actions, and links to validated guidance documents, particularly during the early stages of an outbreak [3,4]. Due to their global scope, public availability, and standardized format as official WHO reports documenting outbreak response activities, DONs were selected as the unit of analysis, as they allow consistent comparison across emergencies and provide a suitable source for assessing the reporting of operational research.

Operational research plays a critical role in informing evidence-based decision-making during public health emergencies; however, important gaps remain in the mechanisms available to communicate, coordinate, and disseminate operational research findings across response efforts [1,2]. To date, no formal evaluation has assessed whether the DONs —the only widely available global reporting mechanism that may indirectly capture operational research activities— or any other research dissemination platform adequately supports operational research needs in emergency contexts. As a result, gaps remain in understanding how operational research is characterized, documented, and communicated within DONs reports and to the public in general.

To address this gap, this study aims to (1) describe trends in key domains of DONs reports, including geographic distribution, temporal patterns, disease types, and response activities; (2) characterize whether and how operational research is referenced within DONs reports; and (3) identify ways in which DONs could be leveraged to better support and disseminate operational research during infectious disease outbreaks.

## Methods

### Study design

This study used a structured content analysis approach combining descriptive statistics with thematic characterization based on primarily predefined, deductive coding domains. The overall process consisted of identifying Disease Outbreak News (DONs) reports that met the inclusion criteria, generating descriptive statistics for key variables of interest, and conducting dual deductive coding using a codebook informed by existing literature. Quantitative results were tabulated, and qualitative codes were organized into themes.

Because this study analyzed secondary data obtained from a publicly available source, it did not require institutional ethical review. The authors determined that the study posed no individual risk or privacy concerns, as all data were derived from publicly accessible documents.

### Materials

Reports published on the World Health Organization (WHO) Disease Outbreak News (DONs) website between January 2012, and October 2024 were compiled, resulting in the identification of 412 outbreak events (N = 412). DONs related to COVID-19 and foodborne pathogens were excluded according to predefined inclusion criteria developed with input from subject matter experts with experience in outbreak response. COVID-19 was excluded because its prolonged pandemic nature did not align with the study focus on acute emergency events. Foodborne pathogens were excluded because their typically shorter duration differs from that of other infectious disease outbreaks included in this analysis.

The selected timeframe, January 2012 through October 2024, was chosen to capture events beginning with the emergence of Middle East respiratory syndrome (MERS), one of the first major epidemics following implementation of the International Health Regulations (2005), and to allow analysis of outbreak response activities through the present period.

### Procedure

Eligible DONs reports were downloaded and stored on a secured, shared platform for analysis. Descriptive statistics were generated to characterize outbreak and response features, including pathogen type, responding country, WHO region, year of outbreak onset, number of reports per outbreak, response pillars involved, and whether operational research was mentioned. Response activities were categorized according to the eight response pillars defined by the Global Outbreak Alert and Response Network (GOARN): surveillance, laboratory and diagnostics, case management or clinical care, infection prevention and control, logistics and coordination, points of entry, vector control, and risk communication and community engagement [6].

Qualitative content analysis was conducted using deductive thematic coding [7]. A coding framework was developed through team discussion, pilot testing, and refinement in accordance with established qualitative analysis methods [8]. Coding definitions and operational criteria were determined using the literature and input from expert opinion, as there are not standard frameworks defining operational research, technical support, and deployment. For the purposes of this study, technical support is defined as guidance documents or support to aid in outbreak response and deployment refers to ongoing activities of outbreak mitigation [9–12]. The final coding framework included the following domains: pathogen, risk communication, surveillance, laboratory and diagnostics, case management, infection prevention and control, points of entry, vector control, outbreak duration, technical support, deployment, operational research, reporting tone, diversity of reporting features, operational research activities, guidance provided, deployment support, and conflicting messaging.

To ensure coding reliability, more than 10% of the sample was independently coded by two investigators until full agreement was achieved, consistent with recommended qualitative research practices [13]. Iterative team review of coded data resulted in the identification of five major themes. Representative excerpts from DONs reports were selected to illustrate key qualitative findings. All analyses were conducted using Microsoft Excel.

## Results

This study examined 412 outbreak events reported in eligible Disease Outbreak News (DONs) reports. Two primary findings emerged: (1) the descriptive characteristics of reported outbreaks and response activities, and (2) the presence of inconsistent terminology across DONs reports. These results highlight gaps in the documentation and communication of response efforts that may limit the ability to promote a comprehensive, evidence-based approach to outbreak response.

**Fig 1:**
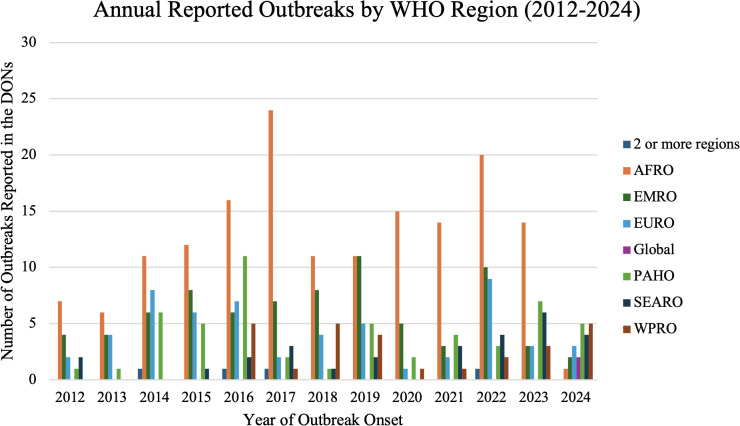
Annual Reported Outbreaks by WHO Region (2012-2024).

Fig 1 shows the number of outbreaks reported each year, categorized by World Health Organization (WHO) region. The highest numbers of DON-reported outbreaks occurred in 2022 (n = 49, 11.9%) and 2016 (n = 48, 11.7%). Overall, 39.3% (n = 162) of reported outbreaks occurred in the African Region. Only six outbreaks (1.45%) involved multiple WHO regions; all were associated with vector-borne diseases, including Zika and yellow fever.

Table 1 summarizes the number of outbreaks reported in each WHO region from January 2012 through October 2024. Table 2 lists the most frequently reported pathogens in the DONs sample, with Middle East respiratory syndrome coronavirus (MERS-CoV) accounting for 13.1% of outbreaks, followed by influenza (12.9%) and poliomyelitis (9.7%).

**Table 1:** Outbreaks by WHO Region as Reported by the DONs (N = 412).

WHO Region	N (%)
AFRO	162 (39.3%)
EMRO	78 (18.9%)
EURO	56 (13.6%)
PAHO	54 (13.1%)
SEARO	28 (6.8%)
WPRO	27 (6.6%)
2 or more regions	6 (1.5%)

**Table 2:** Top 10 Pathogens Across Outbreaks As Reported by the DONs (N = 412).

Pathogen	n(%)
MERS-CoV	54 (13.1%)
Influenza	53 (12.9%)
Polio	40 (9.7%)
Yellow Fever	32 (7.8%)
Cholera	29 (7.0%)
Dengue	23 (5.6%)
Measles	23 (5.6%)
Ebola	19 (4.6%)
Lassa Fever	18 (4.4%)
Chikungunya	12 (2.9%)

**Fig 2:**
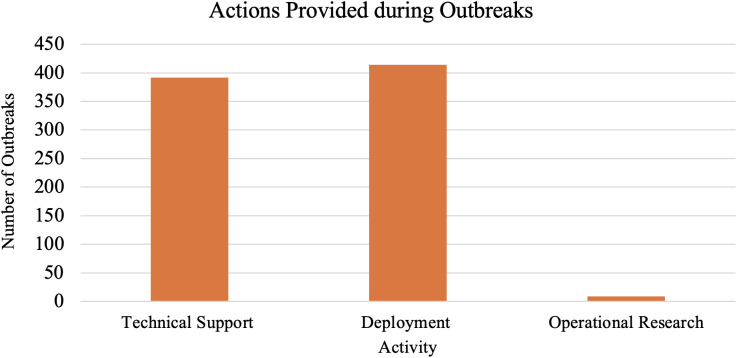
Actions Provided during Outbreaks as Reflected by the DONs.

Fig 2 shows the number of outbreaks in which deployment, technical support, or operational research was reported. Most DONs reports described technical support (n = 392, 95.1%) and all reports documented deployment activities (n = 412, 100%). In contrast, operational research was explicitly mentioned in only 9 reports (2.2%). These findings indicate that although DONs reports provide substantial information on response activities and technical assistance, they rarely document operational research efforts.

**Fig 3:**
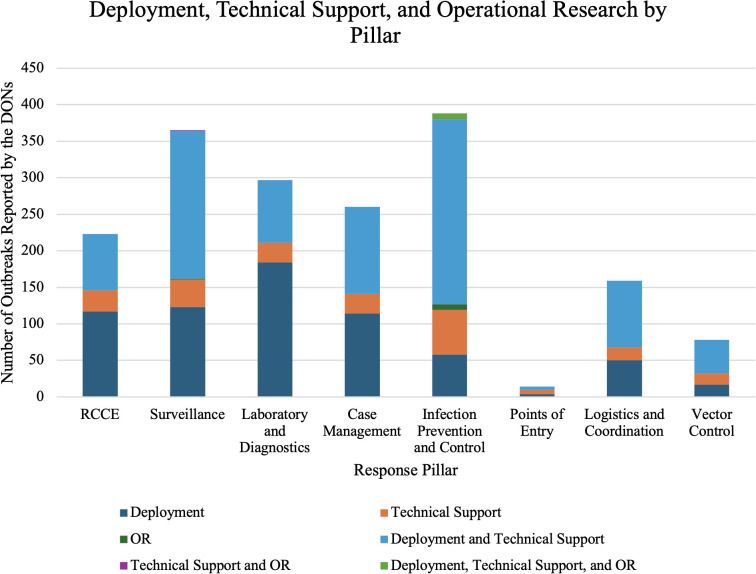
Deployment, Technical Support, and Operational Research by Pillar as Reflected by the DONs.

When categorized by response pillar (Fig 3), 8 of the 9 reports that referenced operational research were related to infection prevention and control, and 1 was related to surveillance. Operational research was explicitly mentioned in reports describing outbreaks of Ebola, cholera, typhoid, Marburg virus disease, yellow fever, chikungunya, and Nipah virus. Among the 9 DONs reports that referenced operational research, 7 involved outbreaks in the African Region, and the remaining 2 involved outbreaks in the South-East Asia Region and the Western Pacific Region. These findings identify the regions, pathogens, and response domains in which operational research was most frequently documented within DONs reports.

In addition to the quantitative results, thematic analysis identified four primary themes (Table 3), including increasing comprehensiveness and standardization of DONs reports over time, the frequent association of deployment with laboratory and diagnostic activities, the frequent association of technical support with infection prevention and control, and the limited scope of operational research references.

**Table 3:** Thematic Findings.

Key Thematic Finding	Example Excerpt from a DON Report
**Increased comprehensiveness and standardization of DONs over time**	*“Description of the situation”(Avian Influenza H5N1, Egypt, 2012).* *“Situation at a glance….description of the situation…epidemiology… public health response…WHO risk assessment [and]…WHO advice” (Diphtheria, Nigeria, AFRO, 2023).*
**Deployments most frequently related to laboratory and diagnostics**	*“An antimicrobial susceptibility test for Vibrio cholerae 01 Ogawa was conducted by the Institute of Epidemiological Diagnostics and Reference” (Cholera, Mexico, PAHO, 2013).*
**Technical support most frequently related to infection prevention and control**	*“WHO advice… Food hygiene practices should be observed. People should avoid drinking raw camel milk… or eating meat that has not been properly cooked” (MERS-CoV, Austria, EURO, 2014).*
**Operational research limited to biomedical interventions**	*“WHO convened an expert meeting to prioritize candidate vaccines… a ring vaccination trial protocol was developed”* *(Ebola, Uganda, AFRO, 2023).*

### Increased comprehensiveness and standardization over time

Over the study period, DONs reports became progressively more comprehensive and structurally standardized. Reports published early in the study period primarily consisted of brief situation summaries describing incidence and prevalence. In later years, reports more consistently included structured components such as situation updates, epidemiologic assessments, risk assessments, and descriptions of WHO response activities across multiple outbreak response pillars. Early reports focused largely on biomedical response activities, particularly laboratory and diagnostic testing and infection prevention and control. In more recent years, reports increasingly documented a broader range of response activities, including logistics and coordination, risk communication and community engagement, and case management. This trend indicates a gradual shift toward more detailed and multidimensional reporting of outbreak response efforts.

### Deployment most frequently related to laboratory and diagnostics

Among reports describing deployment activities, laboratory and diagnostic response functions were the most frequently cited. Overall, 33.1% of deployment activities were related to laboratory and diagnostic support, with many reports emphasizing case detection, specimen testing, and confirmation of infection. This pattern is consistent with the intended purpose of DONs reports, which often document response activities during the early stages of an outbreak, when case identification and laboratory confirmation are critical.

Although laboratory-focused deployments were observed throughout the study period, reports published after the 2014 Ebola epidemic more frequently included references to socio-behavioral response activities, such as risk communication and community engagement, in addition to laboratory support.

### Technical support most frequently related to infection prevention and control

Technical support activities were most commonly associated with infection prevention and control (IPC). Of the 222 instances in which technical support was described, 61 were related to IPC. These reports frequently included guidance on vaccination strategies, hygiene practices, isolation procedures, and other measures intended to reduce transmission. Descriptions of technical support often involved a combination of disseminating best-practice guidance and providing on-the-ground assistance. In many reports, WHO or partner organizations supplied educational materials, technical recommendations, or personal protective equipment to healthcare workers and response teams. Unlike operational research, which seeks to generate new evidence, technical support activities were primarily described as the application of existing knowledge and established best practices to strengthen outbreak response.

### Operational research limited to biomedical interventions

References to operational research were uncommon and were largely limited to biomedical or surveillance-related activities. Only 9 DONs reports explicitly mentioned operational research. These reports described efforts to evaluate strategies intended to improve outbreak response, most frequently related to vaccination, treatment, or surveillance. Examples included testing an Ebola ring vaccination protocol in Uganda, developing candidate vaccines for zoonotic H5N8 influenza in the Russian Federation, and evaluating alternative treatment regimens for gonococcal infection in the United Kingdom. Other reports described the development of research infrastructure, such as establishing a research and development blueprint in response to Nipah virus in India and supporting global antibiotic research partnerships during a typhoid outbreak in Pakistan. Several reports described the use of surveillance or epidemiologic investigations to inform response activities. For example, an investigation of concurrent typhoid, shigellosis, and cholera outbreaks in the Democratic Republic of the Congo examined epidemiologic patterns of intestinal perforation and mortality. Additional reports described entomologic surveys conducted during a Chikungunya outbreak and studies examining the origin of influenza A(H3N8). Only one report, describing an Ebola outbreak in the Democratic Republic of Congo, referenced the use of questionnaires to examine operational and strategic factors such as lessons learned, at-risk populations, and health-seeking behaviors to inform response planning.

Across reports, the terminology used to describe operational research and response activities varied considerably. This variability limited the ability to consistently identify operational research across response pillars and outbreak settings.

## Discussion

This analysis examined 412 outbreak events reported in World Health Organization (WHO) Disease Outbreak News (DONs) reports to better understand how outbreak response activities, including operational research, are documented in this widely used global communication platform. Both quantitative and qualitative findings demonstrated that DONs provide substantial information on outbreak characteristics, response activities, and technical assistance, but rarely document operational research. Across the study period, reports became more comprehensive and standardized, and frequently described deployment and technical support activities across multiple response pillars. However, explicit references to operational research were uncommon and were largely limited to biomedical or surveillance-related interventions. In addition, terminology used to describe response activities varied across reports, making it difficult to systematically identify operational research efforts.

The quantitative findings showed that DONs reports consistently documented core response functions, particularly deployment and technical support, with nearly all reports describing at least one form of external assistance. Deployment activities were most often related to laboratory and diagnostic functions, while technical support most frequently involved infection prevention and control. These patterns are consistent with the primary purpose of DONs, which is to communicate key response actions during the early stages of an outbreak, when case detection, confirmation, and immediate control measures are critical. In contrast, operational research was explicitly mentioned in only a small proportion of reports and was most often associated with vaccine trials, treatment strategies, or surveillance investigations. This limited representation suggests that operational research activities, although likely occurring during many outbreak responses, are not routinely captured in DONs reporting.

Thematic analysis further demonstrated that DONs reports have evolved over time toward greater completeness and structural consistency. Early reports primarily provided brief situation summaries, whereas more recent reports included epidemiologic assessments, risk evaluations, and descriptions of response activities across multiple operational pillars. This trend reflects broader changes in global outbreak response practice, including increased emphasis on coordination, risk communication, logistics, and community engagement in addition to biomedical interventions. Despite this increased comprehensiveness, operational research remained infrequently reported, and when present, was largely restricted to biomedical domains. Few reports described operational research related to program implementation, behavioral interventions, or health systems response, and only one report explicitly referenced the use of research to inform operational decision-making processes.

These findings should be interpreted in the context of the intended purpose of the DONs platform. DONs are designed primarily as an official WHO communication mechanism to inform Member States, partners, and the public about acute public health events and the actions being taken in response. In accordance with the International Health Regulations (2005), DONs serve to provide timely updates on outbreak occurrence, geographic spread, and control measures. As such, DONs are not intended to function as a comprehensive repository of all response activities, nor are they designed to serve as a formal reporting mechanism for operational research. The emphasis on rapid communication, standardized format, and concise reporting may limit the extent to which detailed descriptions of research activities are included. In this context, the limited documentation of operational research observed in this analysis likely reflects the scope and purpose of DONs rather than the absence of such work in the field.

It is also important to acknowledge that DONs are not the only forum in which operational research findings may be communicated. Operational research conducted during outbreaks is often disseminated through peer-reviewed publications, technical briefs, situation reports, after-action reviews, and internal partner reports. These formats may be better suited for detailed methodological description, analysis, and interpretation of findings. In addition, operational research may be documented within partner organizations, academic institutions, or national public health agencies without being reflected in WHO reporting platforms. The absence of operational research in DONs reports therefore does not imply that such activities are not occurring, but rather that they may be communicated through other channels.

Despite these limitations, DONs remain one of the most widely used and influential global communication platforms for acute public health emergencies. DONs reports are publicly available, globally accessible, and published in a standardized format, allowing consistent comparison across outbreaks and regions. They are frequently used by national authorities, international organizations, researchers, and response partners as a primary source of information during rapidly evolving events. Because of their visibility and credibility, DONs play an important role in shaping understanding of outbreak response priorities and practices.

For these reasons, DONs may represent a valuable opportunity to elevate the visibility of operational research during infectious disease outbreaks. Even brief, standardized references to operational research activities within DONs reports could help promote awareness of ongoing studies, facilitate coordination among response partners, and support the dissemination of lessons learned across emergencies. Incorporating consistent terminology, links to available research briefs, or optional reporting fields related to operational research could improve the ability to track how evidence is generated and applied during response efforts, without altering the primary purpose of DONs as a rapid communication tool. Strengthening the integration of operational research within existing reporting platforms may ultimately contribute to more systematic learning across outbreaks and to more effective, evidence-informed emergency response.

Overall, this analysis highlights that DONs reports provide valuable insight into outbreak response activities but currently capture only a limited portion of operational research conducted during emergencies. Improving the consistency of terminology and expanding the visibility of operational research within widely used communication platforms such as DONs may help strengthen the link between response practice and evidence generation, thereby supporting more effective and coordinated infectious disease outbreak response in the future.

### Strengths and limitations

To our knowledge, this study is the first review of WHO Disease Outbreak News (DONs) reports to examine how outbreak response activities are documented and to assess the extent to which operational research is reported within this platform. This analysis covered a 12-year period, allowing evaluation of trends in reporting practices and changes in the documentation of response activities over time. The study included a large sample and used an iterative analytic approach informed by a multidisciplinary team with expertise in outbreak response, clinical medicine, and public health research, strengthening the validity of the coding framework and interpretation of findings.

This study has several limitations. First, the analysis relied on investigator-defined classifications for operational research, technical support, and deployment because standardized definitions are not consistently used in the literature. Although definitions were informed by published sources and expert input, some misclassification may have occurred, particularly when response activities were described briefly or inconsistently. Second, the analysis relied exclusively on DONs reports. Although DONs provide a widely accessible and standardized source of outbreak information, they do not represent a complete record of all response activities, and not all outbreaks are reported through this platform. In addition, DONs are intended to provide timely situational updates rather than comprehensive documentation of response efforts. As operational research may occur later in an outbreak or be reported through other channels, it may not be captured in DONs even when conducted. Nonetheless, because DONs remain a widely used global communication platform during public health emergencies, understanding their scope and limitations is important for identifying opportunities to improve the visibility of operational research during outbreak response.

## Conclusions

This analysis identified gaps in how operational research is documented within WHO Disease Outbreak News (DONs) reports, despite frequent reporting of deployment and technical support activities during infectious disease outbreaks. Although DONs have become more comprehensive and standardized over time, explicit references to operational research were uncommon and were largely limited to biomedical or surveillance-related interventions. These findings suggest that the DONs do not fully capture the role of operational research in outbreak response. Operational research can be documented outside of the DONs in peer-reviewed publications, technical briefs, or internal response documents. Therefore, the absence of operational research within the DONs should not be interpreted as evidence that activities did not occur. Enhancing existing reporting mechanisms, such as incorporating standardized terminology or optional fields related to operational research within DONs guidance, could help strengthen the evidence base describing how operational research informs response efforts. Such changes may also support monitoring of WHO’s Emergency Response Framework, which identifies operational research as a guiding principle of emergency response [12]. Improving the visibility of operational research during public health emergencies may require additional support for consistent documentation and communication of these activities. DONs are not intended to be the sole forum for reporting operational research, and peer-reviewed publications, technical reports, and situation updates remain important dissemination mechanisms. Further work beyond the DONs platform is needed to better understand how and whether operational research is conducted and communicated during outbreaks and how reporting practices can support more effective, evidence-informed emergency response.
